# Emotions Induced by Recalling Memories About Interpersonal Stress

**DOI:** 10.3389/fpsyg.2021.618676

**Published:** 2021-04-09

**Authors:** Sachiyo Ozawa

**Affiliations:** UTokyo Center for Integrative Science of Human Behavior (CiSHuB), Evolutionary Cognitive Sciences, Graduate School of Arts and Sciences, The University of Tokyo, Tokyo, Japan

**Keywords:** autobiographical recall, episodic memory, emotion induction, social emotions, positive and negative affect schedule (PANAS), Russell’s circumplex model, emotional valence and arousal

## Abstract

The emotions that people experience in day-to-day social situations are often mixed emotions. Although autobiographical recall is useful as an emotion induction procedure, it often involves recalling memories associated with a specific discrete emotion (e.g., sadness). However, real-life emotions occur freely and spontaneously, without such constraints. To understand real-life emotions, the present study examined characteristics of emotions that were elicited by recalling “stressful interpersonal events in daily life” without the targeted evocation of a specific discrete emotion. Assuming generation of mixed and complex emotions, emotional groups with relatively strong correlation of multiple emotions according to surprise, fear, anger, disgust, sadness, and happiness were expected. Seventy-two university students (35 males, mean age: 19.69 ± 1.91 years; 37 females, 20.03 ± 2.42) participated in the study. In the emotion induction procedure, participants freely recalled memories as per the instructions on a monitor, and then responded silently to a series of questions concerning any one recalled incident. Assessments of emotional states using emotion scales and another item indicated that validated emotional changes had occurred during the task. Inter-correlations between six emotions demonstrated an emotional group consisting of disgust and anger, which frequently occur as negative interpersonal feelings, and that of fear and sadness. This indicated generation of mixed and complex emotions as experienced in social life. Future studies concerning relationships between these emotions and other factors, including neurophysiological responses, may facilitate further understanding about relationships between mental and physiological processes occurring in daily life.

## Introduction

People experience various emotions in real-life, including mixed and complex ones. When an unpleasant interpersonal event happens in real-life, people may ruminate about it and languish in an unpleasant mood consisting of emotions, such as anger, sadness, and disgust. In fact, emotions occurring in social contexts have been found to be complex and mixed (Burnett et al., 2011; Burkitt et al., 2019). These emotions generally occur due to situations related to mental and physiological well-being, decision-making, and social relationships, and exert constant influence on people’s daily lives.

Autobiographical recall is frequently used as an emotion induction procedure in laboratory settings (e.g., Suardi et al., 2016; Siedlecka and Denson, 2019). This method typically instructs participants to vividly recall episodic memories associated with certain emotions, such as happiness, anger, and sadness, or any other pleasant or unpleasant emotions (e.g., Strack et al., 1985; Damasio et al., 2000; Krauth-Gruber and Ric, 2000; Marci et al., 2007; Gadeikis et al., 2017; Monnier et al., 2018; Jahanitabesh et al., 2019; Nawa and Ando, 2019). A specific discrete emotion (e.g., sadness) is frequently assigned to be induced; however, it has been argued that emotions usually emerge in groups, in various combinations, and with varying degrees of intensity. In other words, they do not occur in isolation (Mills and D’Mello, 2014). Indeed, emotions induced by autobiographical recall can become more complex and intertangled than emotions triggered by external emotional stimuli such as emotional images. Thus, targeting a specific discrete emotion may be less effective in producing emotions similar to those occurring in real-life experiences because in real-life emotions freely emerge and dissipate in any combination, without any constraints.

Autobiographical recall typically employs free recall which depends on participants’ voluntary efforts. It may be difficult depending on individual ability and recall contexts; hence, the act of writing down memories is occasionally employed to facilitate effective recall (Strack et al., 1985; Dunn and Schweitzer, 2005). Nevertheless, writing is not always applicable for certain experimental conditions (e.g., neuroimaging, physiological responses, etc.) which have some constraints (e.g., limited time and physical mobility, etc.). In a previous study, we examined changes in emotions and pupil sizes while recalling “stressful interpersonal events in daily life” to analyze psychophysiological aspects of emotions similar to real-life experiences (Ozawa et al., 2020). Similarly, in the present study, instead of writing, we asked a series of questions concerning the contents of a recalled memory (e.g., “What kind of emotion did you feel?”; “How did you feel about the person?”) on a monitor consistently after free recall and observed changes in pupil size. We found that pupil sizes constricted while responding to questions and the pupil constriction was associated with the increase of subjective distress, indicating that the act of responding to the questions facilitated the generation of emotions.

Because we specified the type of situation to be recalled and not a specific discrete emotion in the emotion induction procedure, induced emotions were expected to be mixed and complex as experienced in real-life in social contexts. However, characteristics of emotions have not been studied yet. Therefore, the present study aimed to examine how much participants experience emotions from six categories (surprise, fear, anger, disgust, sadness, and happiness) and how these emotions are correlated using the same emotion induction procedure as in Ozawa et al. (2020). The six discrete emotions were selected according to prototypical emotional episodes, defined as “what most people consider the clearest cases of emotion” (Russell and Barrett, 1999). Assuming generation of mixed and complex emotions, there were expected to be emotional groups where multiple emotions would be relatively strongly correlated.

Furthermore, we have previously shown the validity of emotional changes by pre- and post-score comparisons for emotional states assessed by emotion scales and an emotion item; however, the sample size was relatively small (20 participants). Therefore, using a larger sample size than Ozawa et al. (2020), the present study also aimed to examine replicability of the validity of emotional changes for investigation of reliability. Moreover, the relationships between changes in these scores and the six emotion scores were examined to investigate characteristics of these tools.

## Materials and Methods

### Participants

The study conformed to the Declaration of Helsinki and was approved by the University of Tokyo (Approval No. 693-2). All participants were recruited through advertisements (e.g., on university bulletin boards) and were preliminarily informed that the experiment would ask them to recall unpleasant events. Only healthy individuals who reported having no history of psychiatric or neurological diseases were recruited. All participants were students from the University of Tokyo. Seventy-two individuals agreed to participate (mean age: 19.86 ± 2.18; 35 males, mean age: 19.69 ± 1.91 years, range: 18–27; 37 females, 20.03 ± 2.42, 18–30).^1^

### The Positive and Negative Affect Schedule

The Positive and Negative Affect Schedule (PANAS) is a widely used self-report measure of emotional states (Watson et al., 1988). The Japanese version of the PANAS (Sato and Yasuda, 2001) was used to evaluate participants’ emotional states during the experiment. It consists of the PANAS Negative Affect (NA) and Positive Affect (PA) scales. The former represents subjective distress and dissatisfaction regarding various negative mood states (Watson et al., 1988), such as jittery, scared, upset, afraid, nervous, distressed, ashamed, and irritable. The latter represents the extent to which a person feels enthusiastic, active, and alert (Watson et al., 1988), and includes the items: active, proud, strong, inspired, determined, excited, alert, and enthusiastic. Items are rated on a six-point scale ranging from 1 (*not at all*) to 6 (*very*). The Japanese versions of the PANAS NA and PA scales were both found to have good internal consistency (Cronbach’s alphas of 0.98 and 0.99, respectively; Sato and Yasuda, 2001).

### Experimental Procedure

**Figure 1:**
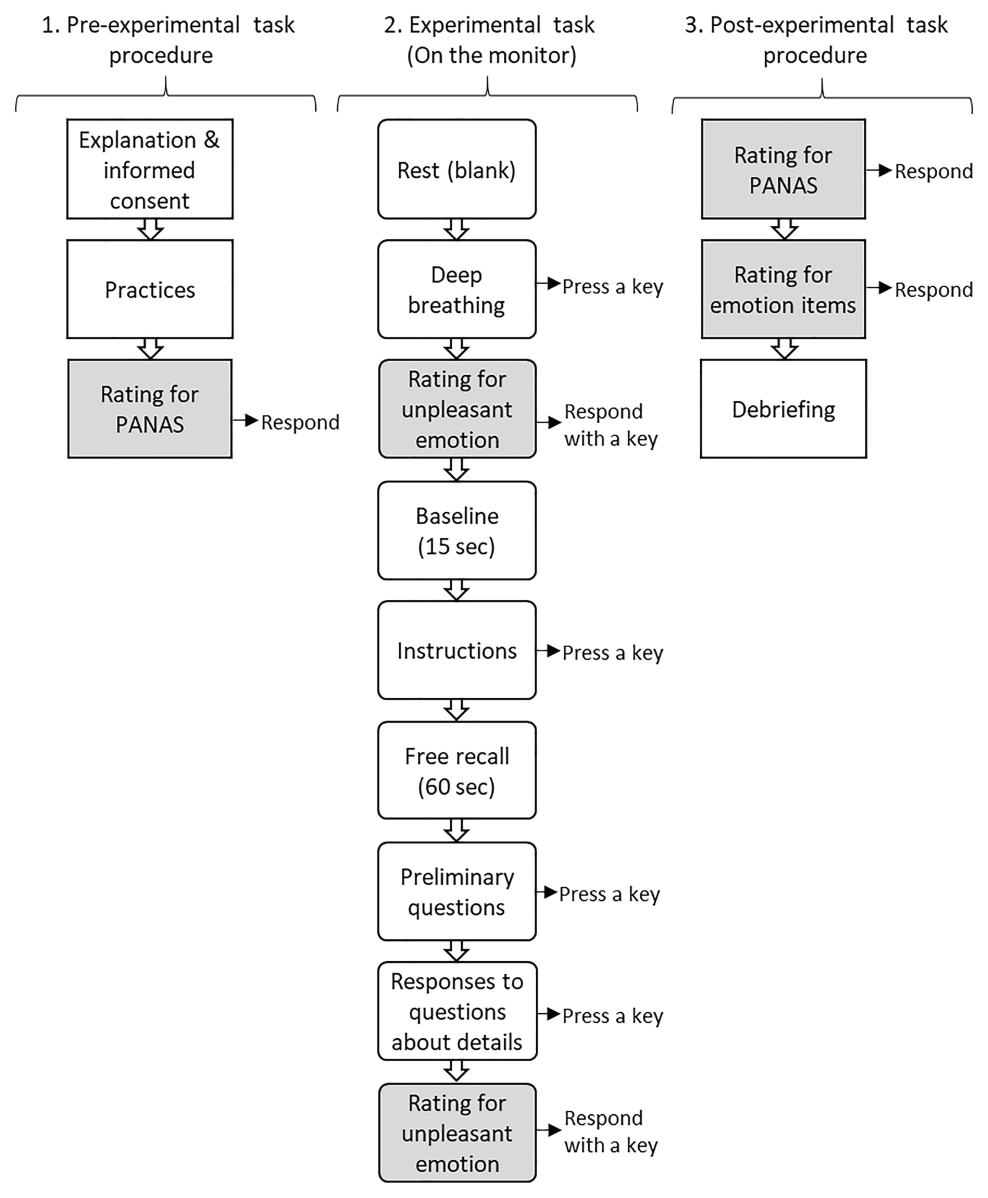
Flow chart of the experimental procedures.

Figure 1 shows overall sequences of the experimental procedure in this study. Initially, participants were given thorough explanations about the experiment, and subsequently provided written informed consent. They were informed that they would recall stressful interpersonal events from their daily life; would not be asked to provide the contents of those memories following the experimental task; and could withdraw from the study at any time, without fear of reprisal or negative consequences. Finally, an experimenter asked about and confirmed their physical and mental conditions at the time of the study to ensure their eligibility for participation.

The experimental task was conducted in a shielded room with dim lighting. Initially, an experimenter instructed participants to conduct a few practice sessions to confirm the experimental task procedure. They were informed that a series of instructions or questions written in white letters would be displayed on the black background of a monitor in the experimental task. Each monitor was offset by the participant’s key press, at their own pace (except baseline and free recall that were set at 15 and 60 s, respectively). Participants were instructed to proceed to the next screen by a key press after fully understanding the previous instructions or adequately responding the relevant questions in their minds. Subsequently, participants completed the PANAS, which measured their emotional state at the time of the experiment.

At the beginning of the experimental task, the screen on the monitor displayed instructions to relax and engage in a deep breathing exercise to control respirational state because respiration has been noted to be associated with emotional state (Homma and Masaoka, 2008). On the next screen, participants responded to a one-item unpleasant-emotion question (“How unpleasant do you feel now?”), measured on a seven-point scale (1: *not at all*, 7: *very*), by pressing a key. A screen for a 15-s baseline was then displayed, with the following instructions: “Take a brief break. Instructions will be displayed soon (wait a while before pressing a key).” Subsequently, a series of instructions were presented (e.g., “You will be asked to recall stressful interpersonal events in daily life as clearly and vividly as possible.”; “you will not be asked for the recalled contents.”). Each instruction was simultaneously presented at the participant’s key press. Next, a free recall was undertaken for 60 s using the following instructions: “Recall while viewing this screen (wait a while before pressing a key).” During the free recall, the participant could recall any number of stressful incidents. After the free recall, the participant was given a few instructions and was asked some questions to help them pick out one stressful incident to focus on in the next step (e.g., “Were you able to remember a stressful experience?”; “When did the incident occur?”; and“You will be asked about the experience shortly.”). Next, a series of questions about the details of the behaviors and emotions of the participants and other persons involved in the event were presented using the following instructions: “What did the person do to you?”; “What did you do to the person?”; “What kind of emotion did you feel?”; “How did you feel about the person?”; “How did you feel about yourself?”; “Did the person know your feelings?”; and “What did you think the person thought about you?.” The series of questions finished with the final instruction “Please recall the emotions you experienced at that time, again.” Next, the unpleasant-emotion item was re-administered. The average time between pre- and post-ratings of the unpleasant-emotion item was 363 ± 126 s, and that of the total experimental task was 433 ± 159 s. E-Prime software (version 2.0; Psychology Software Tools, Sharpsburg, PA, United States) was used to create the experimental task stimulus and to record each participant’s responses.

After the post-rating of the unpleasant-emotion item, participants completed the PANAS to help measure their emotional states. Furthermore, they rated each emotion (“surprise,” “fear,” “anger,” “disgust,” “sadness,” and “happiness”) on a six-point scale (1: *not at all*, 6: *very*) based on the extent to which it was felt while responding to the questions. At the end of the experiment, the experimenter asked about their emotions recalled in the experimental task, without asking about the contents of their memories, as well as the daily life events they found enjoyable (e.g., extracurricular activities, hobbies, and weekend pastimes).^2^

## Results

### Analysis of Ratings for Emotion Items

**Figure 2:**
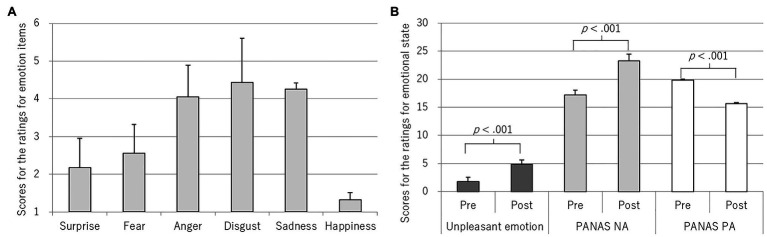
Rating scores. **(A)** Ratings for emotion items. **(B)** Ratings for emotional state.

The mean and SDs for the emotion-item scores are shown in Table 1; Figure 2. No significant gender differences were found in these values. The items “disgust” (4.43 ± 1.25), “sadness” (4.25 ± 1.45), and “anger” (4.06 ± 1.27) had the highest scores, followed by “fear” (2.56 ± 1.50) and “surprise” (2.18 ± 1.33). “happiness” (1.32 ± 0.62) had the lowest score. A one-way repeated measures analysis for these scores showed main effect of emotion items [*F*(5, 355) = 88.76, *p* < 0.001, *η*_p_^2^ = 0.56]. Disgust, sadness, and anger scored higher than fear (*p* < 0.001; *d* = 1.34, 1.15, 1.07), surprise (*p* < 0.001; *d* = 1.74, 1.49, 1.45), and happiness (*p* < 0.001; *d* = 2.87, 2.33, 2.49), and fear and surprise scored higher than happiness (*p* < 0.001; *d* = 0.95, 0.75).

**Table 1:** Means and SDs for rating for emotion items, and their inter-correlations.

	Scores	Inter-correlations					
Emotion items	*M(SD)*	Surprise	Fear	Anger	Disgust	Sadness	Happiness
Surprise	2.18 (1.33)	–	–	–	–	–	–
Fear	2.56 (1.50)	0.35^**^	–	–	–	–	–
Anger	4.06 (1.27)	0.27^*^	−0.11	–	–	–	–
Disgust	4.43 (1.25)	0.26^*^	0.12	0.48^**^	–	–	–
Sadness	4.25 (1.45)	0.32^**^	0.40^**^	0.00	0.12	–	–
Happiness	1.32 (0.62)	0.05	0.00	−0.08	−0.03	−0.04	–

* *p* < 0.05;

** *p* < 0.01.

*N* = 72.

To investigate relationships among emotions, inter-correlations were examined: the item “surprise” was positively correlated with the items of “fear” (*r* = 0.35, *p* = 0.003), “anger” (*r* = 0.27, *p* = 0.021), “disgust” (*r* = 0.26, *p* = 0.029), and “sadness” (*r* = 0.32, *p* = 0.006). The item “fear” was positively correlated with “sadness” (*r* = 0.40, *p* < 0.001), and similarly, the item “anger” with “disgust” (*r* = 0.48, *p* < 0.001).

### Analysis of Ratings for Emotional State

Table 2 shows the means, SDs, and Cronbach’s alphas for the unpleasant-emotion item and the PANAS NA and PA scales in the pre- and post-measurement periods, and difference values calculated by subtracting pre-scores from post-scores. No significant gender differences were found in these values. The unpleasant-emotion item had significantly higher post-scores compared to the pre-scores, *t*(71) = 16.34, *p* < 0.001, *d* = 1.93 (Table 2; Figure 2). The PANAS NA and PA scales showed significant changes in the post-scores relative to the pre-scores; NA: *t*(71) = 7.59, *p* < 0.001, *d* = 0.90, PA: *t*(71) = −6.53, *p* < 0.001, *d* = 0.77.

**Table 2:** Descriptive data for the ratings for emotional state, their pre- and post-comparisons, and difference values.

			Pre		Post		*t* test		Difference values
Rating for emotional state		*N*	*M (SD)*	*α*	*M (SD)*	*α*	*t*	*df*	*M (SD)*
Unpleasant emotion		72 (35)	1.76 (1.26)	–	4.90 (1.46)	–	16.34^*^	71	3.14 (1.63)
PANAS NA		72 (35)	17.18 (6.88)	0.88	23.25 (8.36)	0.87	7.59^*^	71	6.07 (6.79)
PANAS PA		72 (35)	19.81 (5.73)	0.82	15.63 (5.55)	0.81	−6.53^*^	71	−4.18 (5.43)

*N* value in parenthesis represents number of males.

* *p* < 0.001.

### Correlations Between Emotional Changes and Emotion Items

To examine the emotional concepts represented by ratings for emotional state, correlations between the difference values for emotional state and emotion-item scores were examined (Table 3): the difference values for the unpleasant-emotion item were positively correlated with “anger” (*r* = 0.32, *p* = 0.007) and “sadness” (*r* = 0.27, *p* = 0.024), and negatively with “happiness” (*r* = −0.25, *p* = 0.033). Those for the PANAS NA were positively correlated with “fear” (*r* = 0.28, *p* = 0.018) and “sadness” (*r* = 0.37, *p* = 0.002); and those for PANAS PA correlated negatively with “fear” (*r* = −0.28, *p* = 0.019) and “sadness” (*r* = −0.41, *p* < 0.001).^3^

**Table 3:** Correlations between difference values of rating for emotional state and emotion-item score.

Rating for emotional state	Surprise	Fear	Anger	Disgust	Sadness	Happiness
Unpleasant emotion	0.02	0.00	0.32^**^	0.14	0.27^*^	−0.25^*^
PANAS NA	0.18	0.28^*^	−0.04	0.00	0.37^**^	−0.10
PANAS PA	−0.21	−0.28^*^	0.23	0.18	−0.41^**^	−0.12

* *p* < 0.05;

** *p* < 0.01.

*N* = 72.

## Discussion

Participants reported that the emotions of disgust, sadness, and anger were felt the most prominent, followed by fear and surprise (Table 1; Figure 2). Happiness was scarcely felt. The order of the extent of reported emotions appears to be related to emotional mapping represented in circumplex model of Russell (1980), where each emotion is placed according to their emotional arousal and valence. Based on this model, emotional arousal is higher from surprise, fear, anger, disgust, and sadness, in descending order (Russell and Barrett, 1999). Disgust, sadness, and anger are located closely at the moderate-arousal level, whereas, fear and surprise are also located closely, but at the high-arousal level. Happiness is placed at the moderate-arousal level, but unique in positive dimension. Thus, participants felt various emotions, but most likely emotions at the moderate-arousal level in the negative dimension.

With regard to relationships between these emotions, surprise was unique in associating with all the other emotions in the negative dimension. Notable correlations were found between disgust and anger, and sadness and fear. According to the former association, disgust and anger are positioned side-by-side in model of Russell (1980), indicating similar characteristics. In fact, anger and disgust (in addition to contempt) have both been considered as moral emotions that frequently occur as negative interpersonal feelings, as suggested by social-functionalists (Hutcherson and Gross, 2011). Moreover, they have both been considered to involve aggression in response to threats to the self, although anger is the direct aggression that motivates one to approach targets, whereas, disgust is indirect aggression that motivates one to increase distance from sources of this feeling (Molho et al., 2017). Thus, these emotions might be experienced together as mixed emotions, where each might occur simultaneously or sequentially (Oceja and Carrera, 2009). The association of sadness and fear also indicates comorbidity as mixed emotions. Nevertheless, sadness and fear were less likely to be accompanied with disgust and anger, based on their low and non-significant correlations. Thus, the emotion induction method in the present study induced two major emotional groups consisting of multiple emotions, as supposed, indicating existence of mixed and complex emotions.

The pre- and post-score comparison of the PANAS NA or PA and unpleasant-emotion item indicated validated emotional changes. The effect sizes were almost the same as our previous experiment in Ozawa et al. (2020) (PANAS NA, *d* = 0.60; PANAS PA, *d* = 0.57; and unpleasant-emotion item, *d* = 1.83). Thus, this method indicated replicability and reliability. Regarding relationships between these tools and the six emotions, increase in the PANAS NA and decrease in the PANAS PA were associated with the extent of sadness and fear (Table 3). Thus, these scales were especially reflective of the emotional group of sadness and fear, although the PANAS NA scale is known to assess subjective distress regarding various negative mood states (Watson et al., 1988). On the contrary, an increase in an unpleasant emotion was associated with multiple emotional groups (increase in anger and sadness, and a decrease in happiness). That may be because the meaning of “unpleasant” would differ depending on individual episodes. Notably, the unpleasant-emotion item was also unique in involving assessment of anger, which is the important emotion in the present context.

Finally, recalling stressful interpersonal events from daily life generated emotional groups consisting of multiple discrete emotions, indicating generation of mixed and complex emotions. These complex emotions would be similar to those experienced in daily social life (Burnett et al., 2011; Burkitt et al., 2019), and would be continuously occurring and affecting our life experience through various aspects including mental and physiological states. Knowing how to manage or control these emotions is important for mental and physiological well-being. Therefore, there is a need to investigate the characteristics and mechanisms of these complex emotions, and devise effective strategies to control them. Future studies concerning relationships between these emotions and other factors, including neurophysiological responses, may facilitate the understanding of such processes.

### Limitations and Future Directions

One may argue that not specifying an emotion in the instructions could generate confounds with background variables, and may not be suitable for basic research that aims to investigate the basic mechanism of emotional processing. However, as previously discussed, it could induce mixed emotions that are similar to real-life experiences; such examination would be important to understand human psychological processes further. Second, the unpleasant-emotion item is one-item and can quickly assess emotional states, and would be useful in some experimental conditions. However, its reliability is not robust and needs to be carefully interpreted. Finally, this study did not employ a neutral condition (e.g., asking participants to recall a topic or daily event that is neutral in emotional valence). However, this experimental task was developed primarily for examination of relationships between these emotions and other factors, including neurophysiological responses, where emotional arousal is the significant factor. Therefore, comparison of other emotional valence conditions may not be emphasized in the present study unless emotional arousal can be controlled using recall of episodic memories.

## Data Availability Statement

The raw data supporting the conclusions of this article will be made available by the author, without undue reservation.

## Ethics Statement

The studies involving human participants were reviewed and approved by The University of Tokyo. The patients/participants provided their written informed consent to participate in this study.

## Author Contributions

SO developed study concept, performed data collection and analysis, and prepared the manuscript.

### Conflict of Interest

The author declares that the research was conducted in the absence of any commercial or financial relationships that could be construed as a potential conflict of interest.

## Footnotes

^1^*A priori* analysis for *t* tests with matched pairs (G*Power, alpha error probability = 0.05) indicated that this sample size affords 95% power to detect a medium-sized effect (*d* = 0.5). Furthermore, a *post hoc* analysis calculated for the correlations between “disgust” and “anger” (*r* = 0.48) indicated that this sample size affords 99.2% power; similarly, those between “sadness” and “fear” (*r* = 0.40) at this sample size have a 94.3% power.

^2^These questions were given to facilitate expression of emotions because it has been shown to have positive effects in mental and physical health (Pennebaker and Oheeron, 1984) through emotional discharge, known as “catharsis,” as well as cognitive changes with regard to the events by the reconstruction of their psychological meaning, known as “cognitive reappraisal” (Gross, 1998). Questions about enjoyable daily life events were given to shift the participants’ attentional focus to more pleasant memories from relevant unpleasant memories, known as “emotional distraction” (Nolen-hoeksema and Morrow, 1993).

^3^All aforementioned analyses were conducted using IBM SPSS Statistics 27.0 (IBM Corp., Armonk, NY, United States).
